# Ryanodex Reduces Persistent Hippocampal Effects of Single Mild Traumatic Brain Injury in Rats

**DOI:** 10.1177/08977151251366069

**Published:** 2025-10-15

**Authors:** Reed Berlet, Isabel Bear, Cassidy Kessinger, Eliana Whitcomb, Veron Browne, Vinolia Chellaraj, Julian Bailes, John McDaid

**Affiliations:** ^1^ Department of Neurosurgery, Endeavor Health, Evanston, Illinois, USA.; ^2^ Eagle Pharmaceuticals, Inc., Woodcliff Lake, New Jersey, USA.

**Keywords:** traumatic brain injury, ryanodine receptors, calcium dysregulation, neurodegeneration

## Abstract

Traumatic brain injury (TBI) affects millions of individuals annually, with a 53% increase in emergency visits since 2006. Despite its prevalence, no FDA-approved treatments exist to mitigate brain damage or promote recovery. While repeated TBI is widely studied, even a single impact can cause persistent neurological changes, likely mediated by Ca^2+^ dysregulation via effects on ryanodine receptors. In a rat model of mild TBI, using a single closed-head controlled cortical impact, we observed no changes in the levels of hippocampal glial fibrillary acidic protein and phosphorylated tau—both markers of cellular damage. However, mild TBI (mTBI) significantly enhanced synaptic transmission at hippocampal CA3-CA1 synapses and increased CA1 pyramidal cell excitability for at least 30 days—effects that were significantly attenuated by acute and subacute injection of Ryanodex, a concentrated nanocrystalline formulation of the ryanodine receptor allosteric modulator dantrolene. Ryanodex may, therefore, offer a promising intervention to reduce persistent hippocampal dysfunction following mTBI, with potential clinical applications for acute TBI treatment.

## Introduction

Traumatic brain injury (TBI), especially repeated TBI, has become a widely accepted risk factor for several neurodegenerative conditions, including chronic traumatic encephalopathy (CTE) and Alzheimer’s Disease (AD).^1^ The most common causes of TBI-related injuries are motor vehicle crashes and falls, single events that affect people of all ages and backgrounds.^2^ TBI can affect all areas of the brain, but the effects of TBI on more susceptible brain regions such as the hippocampus and cortex can result in deficits in memory, mood, and cognition, which may be highly detrimental to everyday functioning.^1,3^ While a number of different types of medications are used to treat TBI, including sedatives and anticonvulsants, none are specifically FDA-approved for the acute treatment of TBI,^4^ and there is still an unmet need to prevent the long-term neurodegenerative effects of TBI, which may require treatment to be initiated in the emergency room.

A large number of synaptic and cellular events occur acutely after a TBI, including increased pre- and postsynaptic influx of extracellular calcium (Ca^2+^), which can be further amplified by the process of Ca^2+^-induced Ca^2+^ release, mediated though activation of ryanodine receptors (RyRs), located both pre- and postsynaptically on the endoplasmic reticulum (ER) (Fig. 6).^5^ Activation of pre- and postsynaptic RyRs in healthy neurons results in Ca^2+^-mediated downstream effects such as neurotransmitter release, synaptic plasticity, and other cellular processes crucial for synaptic function.^6,7^ RyRs serve as one of the gatekeepers and regulators of intracellular Ca^2+^, and RyR dysfunction has been implicated in many aspects of the intracellular Ca^2+^ dysregulation that occurs in conditions such as malignant hyperthermia, cardiac arrhythmia, diabetes, AD, Parkinson’s disease, and other neurodegenerative diseases.^8–13^

Previous studies have elucidated the cellular pathophysiology of single and repeated TBIs and described the resulting long-term dysfunction in Ca^2+^ signaling and hyperexcitability in hippocampal neurons. For example, 30 days after impact, a single mild TBI (mTBI) results in increased resting cytosolic Ca^2+^ levels and voltage-gated Ca^2+^ channel (VGCC) responses to depolarization of hippocampal CA1 pyramidal cells.^14^ While the physiological basis for these changes is unclear, alterations in Ca^2+^ homeostasis lasting up to 30 days after impact have also been observed, which may affect the ability to buffer further increases in cytosolic Ca^2+^.^15^ RyR dysfunction is associated with increased resting cytosolic Ca^2+^, possibly due to depletion of ER Ca^2+^, in both *in vitro* and *in vivo* models of TBI.^14,16^ This effect of TBI may be due to modulation of RyRs, rendering them “leaky” and is thought to be due to upregulated kinase activity and oxidative sensitization by reactive oxygen species, common results of head injury.^17,18^ As such, modulation of RyRs activity in early TBI may mitigate downstream effects, in addition to providing valuable insight into their role in TBI and related conditions.

Ryanodex (dantrolene sodium) is a drug that stabilizes and prevents aberrant activation of RyRs.^19,20^ It is FDA-approved for the treatment of malignant hyperthermia and the prevention of malignant hyperthermia in patients at high risk. Malignant hyperthermia is a hypermetabolic condition in which mutated RyRs release Ca^2+^ within skeletal muscle fibers, sometimes leading to severe symptoms such as high body temperature, hypercarbia, tachycardia, tachypnea, acidosis, muscle rigidity and, if untreated, potentially death.^21^ Intravenous dantrolene administration decreases mortality rates from malignant hyperthermia from approximately 80% to less than 5%.^22^ Given the potential role of RyR activation in TBI, treatment with Ryanodex in the immediate post-concussive period may attenuate the structural and cellular remodeling that occurs after mTBI and possibly abrogate the clinical symptoms. Ryanodex is an injectable nanosuspension formulation of dantrolene sodium that represents a significant advancement in drug delivery due to its rapid reconstitution, high concentration (250 mg per vial), and improved solubility profile.^23^ Unlike traditional formulations (e.g., Dantrium, Revonto), which require reconstitution with large volumes of sterile water and prolonged preparation times, Ryanodex is composed of nanometer-sized particles suspended in a lyophilized matrix that can be reconstituted with just 5 mL of sterile water in under 1 min.^23^ This allows for rapid intravenous administration, which is particularly advantageous in the context of acute TBI where timing of intervention is critical to minimize secondary neuronal damage. Dantrolene has been shown to inhibit intracellular Ca^2+^ dysregulation and ER stress—both of which are implicated in neuronal cell death following TBI.^5^ The enhanced pharmacokinetic properties of Ryanodex, including faster onset of action and reduced fluid load, make it a preferable option in acute neurotrauma settings, where minimizing cerebral edema and ensuring timely neuroprotective intervention are essential.^19^ Therefore, the Ryanodex nanosuspension not only improves drug handling but is a vital consideration in an emergency setting after TBI. As persistent synaptic and cellular deficits may result from both acute and subacute effects of mTBI on Ca^2+^ signaling, possibly involving RyR activation and/or dysfunction, we investigated whether acute Ryanodex treatment can mitigate the persistent pathophysiological effects of a single mTBI on hippocampal synaptic and cellular circuitry in a rat model.

## Materials and Methods

### Animals

**FIG. 1.**
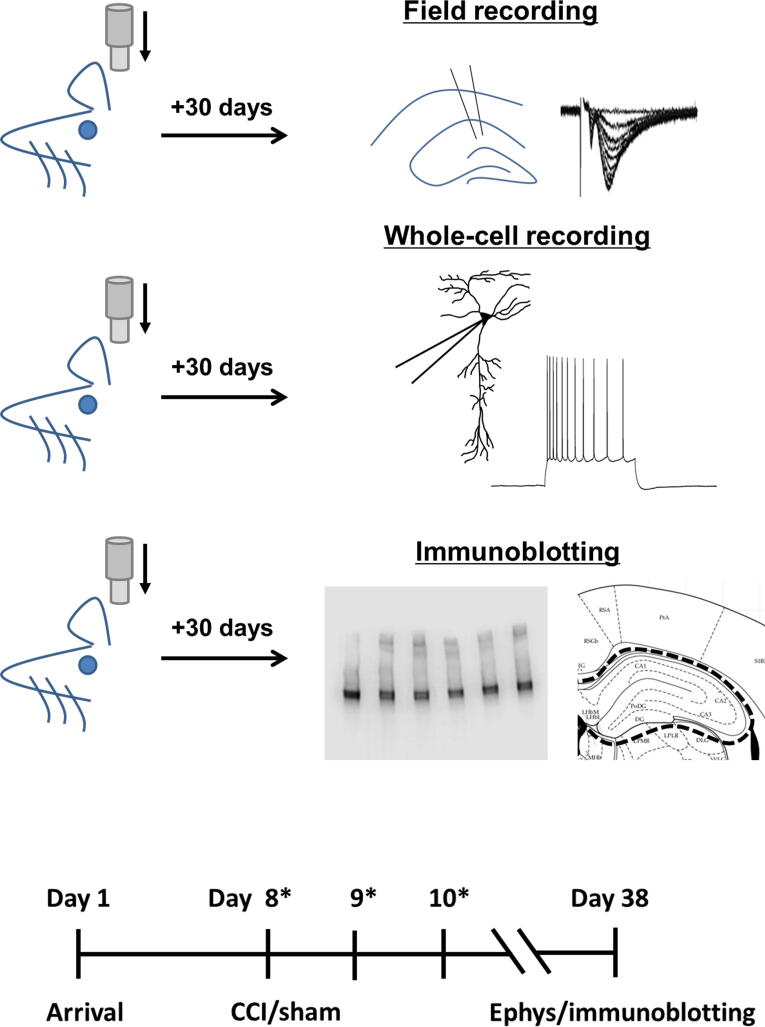
Experimental protocol and timeline. Rats received a single mild CCI. Outcomes were assessed 30 days after impact, for changes in hippocampal synaptic strength using hippocampal CA1 field recordings and hippocampal CA1 pyramidal cell excitability using whole-cell recording (Ephys), in acute brain slices. The hippocampus ipsilateral to the impact site was also assessed for changes in levels of GFAP and phosphorylated tau, using immunoblotting of acutely dissected tissue, see dotted line for tissue dissection border. *Indicates saline or Ryanodex injection (10 mg/kg intravenous). CCI, controlled cortical impact; GFAP, glial fibrillary acidic protein.

Male Sprague-Dawley rats (Charles River Laboratory; 200–250 g) were housed in pairs in the animal facility. They were kept on a 12:12 light/dark cycle, with food and water provided *ad libitum* and were allowed to acclimate to their environment from their arrival (Day 1) through Day 7. On Day 8, rats received either a controlled cortical impact (CCI) or sham procedure, and either Ryanodex (Dantrolene Sodium) (10 mg/kg) or saline, given intravenously 20 min after impact. Rats received Ryanodex injections again on Days 9 and 10. All intravenous injections were performed under isoflurane anesthesia. Thirty days after the CCI or sham procedure, electrophysiology or tissue dissection for immunoblotting was performed (see experimental timeline in Fig. 1). All experiments were performed per the National Institutes of Health Guide for the Care and Use of Animals, and were approved by the Endeavor Health Institutional Animal Care and Use Committee.

### Closed-head controlled cortical impact

To model mTBI, we used a modified CCI approach, as described previously.^14,20^ Rats were anesthetized using isoflurane (Piramal) and placed in a stereotaxic frame, without being restrained by ear bars or an incisor bar (Kopf), on a foam bed. Anesthesia was maintained using a modified nose cone attached to the stereotaxic frame. For analgesia, animals that received a CCI were given subcutaneous ketofen (5 mg/kg), the head was shaved, and topical lidocaine ointment was applied to the scalp prior to the impact. A Leica Impact One stereotactic device (Leica Microsystems) was angled so that the flat impactor tip was perpendicular to the head and the impact was administered using a 5 mm flat tip, at 6 m/sec, at a depth of approximately 2 mm from the surface of the skin covering the right sensorimotor cortex. The impact did not result in any noticeable damage to the overlying skin. Previous studies have established that this experimental setup provides a level of impact that does not result in a skull fracture, but does cause behavioral impairment.^20^ Body temperature was kept at 37°C during recovery, after which rats were placed back in their home cages and monitored daily.

### Brain slice preparation

For field and whole-cell patch recordings, 30 days after a single impact, rats were decapitated under deep isoflurane anesthesia. Brains were removed and transferred into ice-cold *N*-methyl-d-glucamine (NMDG) solution, containing (in mM): 12 *N*-acetyl-cysteine, 93 NMDG, 2.5 KCl, 1.2 NaH_2_PO_4_, 30 NaHCO_3_, 20 4-(2-hydroxyethyl)-1-piperazineethanesulfonic acid, 25 glucose, 5 sodium ascorbate, 1.97 thiourea, 3 sodium pyruvate, 10 MgSO_4_(7H_2_0), 0.5 CaCl_2_ (2H_2_0); pH 7.4 with HCl; bubbled continuously with 95% O_2_/5% CO_2_). Coronal slices (400 μm) containing the ventral hippocampus were obtained using a vibrating blade microtome (VT1000S, Leica Biosystems) in NMDG solution. Slices were transferred to a holding chamber containing artificial cerebrospinal fluid (aCSF) and allowed to recover at room temperature. Standard aCSF solution contained the following (in mM): 125 NaCl, 2.5 KCl, 2 CaCl_2_, 1.2 MgSO_4_, 1.25 NaH_2_PO_4_, 25 NaHCO_3_, 10 d-dextrose equilibrated with 95% O_2_ and 5% CO_2_ (pH 7.3–7.4). Field excitatory postsynaptic potentials (fEPSPs) were recorded using recording microelectrodes (2–6 MΩ) filled with aCSF. Microelectrodes were pulled from borosilicate glass capillaries (Harvard Apparatus) on a P-2000 pipette puller (Sutter Instruments). Synaptic fEPSP responses were evoked in the hippocampal CA1 subfield in response to stimulation of CA3-CA1 Schaffer collaterals, using a bipolar stimulating electrode (World Precision Instruments), with the fEPSP slope calculated as the change in potential (ΔV) of the initial fEPSP waveform over time (t), or ΔV/t (mV/msec). For measurement of synaptic strength, input-stimulus/output-fEPSP slope (I-O) curves were created by plotting the fEPSP slope versus stimulus intensity, using stimulus intensities ranging from 0 μA to 1,000 μA. For measurement of the comparative presynaptic component of the field potential, paired-pulses of evoked fEPSPs were obtained using a 50 msec interstimulus interval. Data was acquired using a MultiClamp 700B amplifier, a Digidata 1440A interface, and Clampex 10.6 software (Molecular Devices). Action potential spike train responses to current injection were obtained in current-clamp mode, acquired at 10 kHz on a MultiClamp 700B amplifier and a Digidata 1440A interface, and Clampex 10.6 software (Molecular Devices). Field and whole-cell data were analyzed using Clampfit 10.6 (Molecular Devices) and Prism 10.0 (Graphpad) software. Input resistance was calculated based on Ohms law (V = IR) using the cell potential response to current injection in the absence of action potential spiking.

### Tissue homogenization

Immunoblotting of the ventral hippocampus ipsilateral to the impact side was performed 30 days after impact to assess changes in levels of glial fibrillary acidic protein (GFAP) and phosphorylated tau, as a measure of hippocampal cell damage. Rats were deeply anesthetized using isoflurane and brains were removed and placed in ice-cold NMDG solution. The ventral hippocampus was acutely dissected out with a scalpel blade, from both the ipsilateral (right) and contralateral (left) sides of a 2 mm coronal slice (−2.5 to −4.5 mm back from bregma, for consistency obtained using a metal brain matrix, Zivic Instruments, see Fig. 1 for dissection border). Acutely dissected tissues were then snap-frozen on dry ice. Tissue homogenates were prepared using a Dounce tissue homogenizer with 15X volume of lysis cell extraction buffer (Invitrogen) mixed with one protease phosphatase inhibitor tablet (Thermo Scientific) per 10 mL of buffer. The buffer volumes needed for homogenization were calculated based on average hippocampal tissue weights. Bolt Lithium dodecyl sulphate Sample Buffer (4X) (Invitrogen) was added (0.33 x lysis volume, μL) to the tissue homogenate. Samples were heated in a dry bath at 70°C for 10 min, then vortexed and kept at −80°C until use.

### Immunoblotting

Prior to SDS-PAGE, samples were placed in a 70°C dry bath for 10 min. Bolt precast 4–12% Bis-Tris Plus gels (Invitrogen) were rinsed under deionized water (diH**_2_**O) and placed in gel electrophoresis tanks with 400 mL Bolt MES SDS running buffer. Thawed samples (10 μL) were vortexed before being loaded onto gels. After electrophoresis at 200 V for 20 min, the gel cassette was rinsed under diH**_2_**O, and placed in diH**_2_**O. Gels were placed onto a PVDF transfer stack (Invitrogen) and separated proteins were transferred to a membrane using the Power Blotter (Invitrogen) at 10 V/1.3 A for 10 min. Membranes were blocked in 20 mL Tris-buffered saline with Tween 20 (TBST) blocking buffer (0.2 g KCl, 3.0 g Tris Base, 500 μL Tween-20, 1.0 g skimmed milk powder) for 1 hour on a tilting table, then washed in TBST for 25 min at room temperature; the TBST was changed every 5 min. Anti-GFAP or anti-phosphorylated tau-181 primary antibody (Abcam) was diluted using 20 mL fresh blocking buffer and incubated with the membrane at 4°C overnight. The membrane was then washed with TBST for 15 min, then incubated with 4 μL of secondary antibody (goat anti-rabbit IgG horse radish peroxidase conjugate; Thermo Fisher) in fresh blocking buffer for 1 hour at room temperature, before washing again with TBST. A chemiluminescent substrate (Pierce ECL) was mixed in equal parts for a total of 4 mL per blot; 2 mL of the substrate was distributed equally on the membrane and left for 10 min. Remaining substrate was spread directly onto a CDigit Blot Scanner (Li-Cor Biotech). The membrane was placed protein-side down onto the scanning area, rolled, and scanned for 12 min using high sensitivity detection.

For assessment of total protein, the membrane was stained using Amido Black Staining Solution 2X (Sigma) prepared at a 1:1 ratio with diH_2_O. The stain was applied for 1 min to visualize total protein content. After staining, the excess dye was carefully discarded, and the membrane was destained using a solution composed of 2 mL acetic acid, 5 mL isopropanol, and 13 mL diH_2_O. The membrane was placed on a tilt table and gently rocked for 30 min to ensure thorough destaining. Following this, the stained protein was scanned using a CanoScan 8600F scanner and saved as an image file. The digitized image was then analyzed using UN-SCAN-IT software (Silk Scientific) to quantify total protein. Densitometric values for the protein of interest were normalized to total protein levels for each sample.

### Statistical analysis

Statistical differences among groups were obtained using a *t*-test or analysis of variance (one-way ANOVA or two-way repeated-measures ANOVA), with Dunnett’s *post hoc* tests where appropriate, using Prism software (GraphPad). Data are shown as mean ± standard error of the mean. Statistical significance was set at *p* < 0.05.

## Results

In this study employing electrophysiology and immunoblotting techniques, we investigated the role of RyRs in the persistent pathophysiological effects of a single mTBI on hippocampal synaptic and cellular function.

### A single mTBI does not affect levels of hippocampal GFAP or phosphorylated tau

**FIG. 2.**
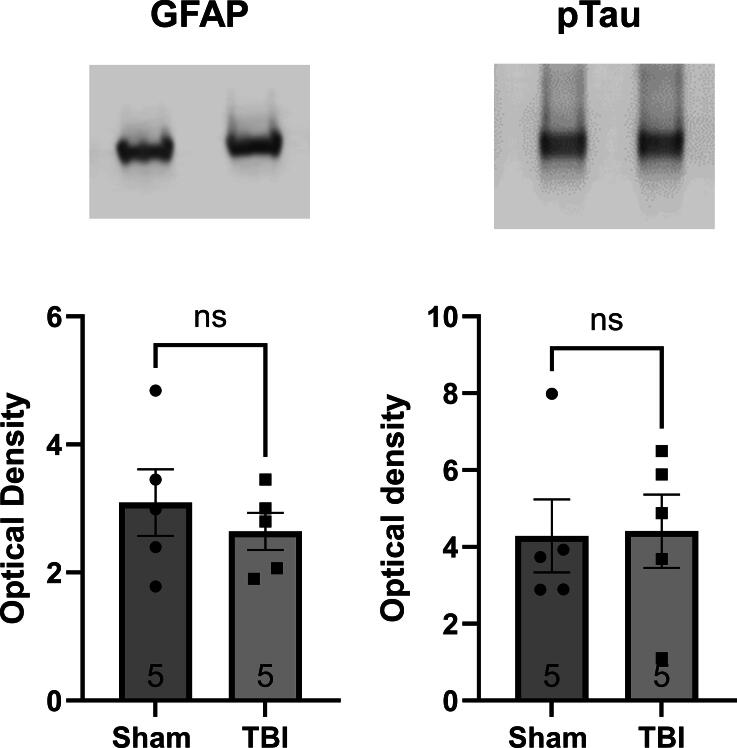
A single mild TBI does not affect hippocampal GFAP or phosphorylated tau levels. Western blot analysis of GFAP and phosphorylated tau, in acutely dissected ventral hippocampus, ipsilateral to the impact site, revealed no effect of a single TBI at 30 days. ns, not significant; GFAP, glial fibrillary acidic protein; TBI, traumatic brain injury.

A single mild TBI had no significant effect on two measures of cell toxicity in ventral hippocampus, on the side ipsilateral to the impact. GFAP levels, when corrected for loading, were similar in the sham and TBI groups (*t*-test; F (4, 4) = 3.20, *p* = 0.47). Similarly, TBI had no effect on phosphorylated tau-181 levels (*t*-test; F (4, 4) = 1.0, *p* = 0.93) (Fig. 2).

### Ryanodex prevents mTBI-mediated increases in hippocampal synaptic strength

**FIG. 3.**
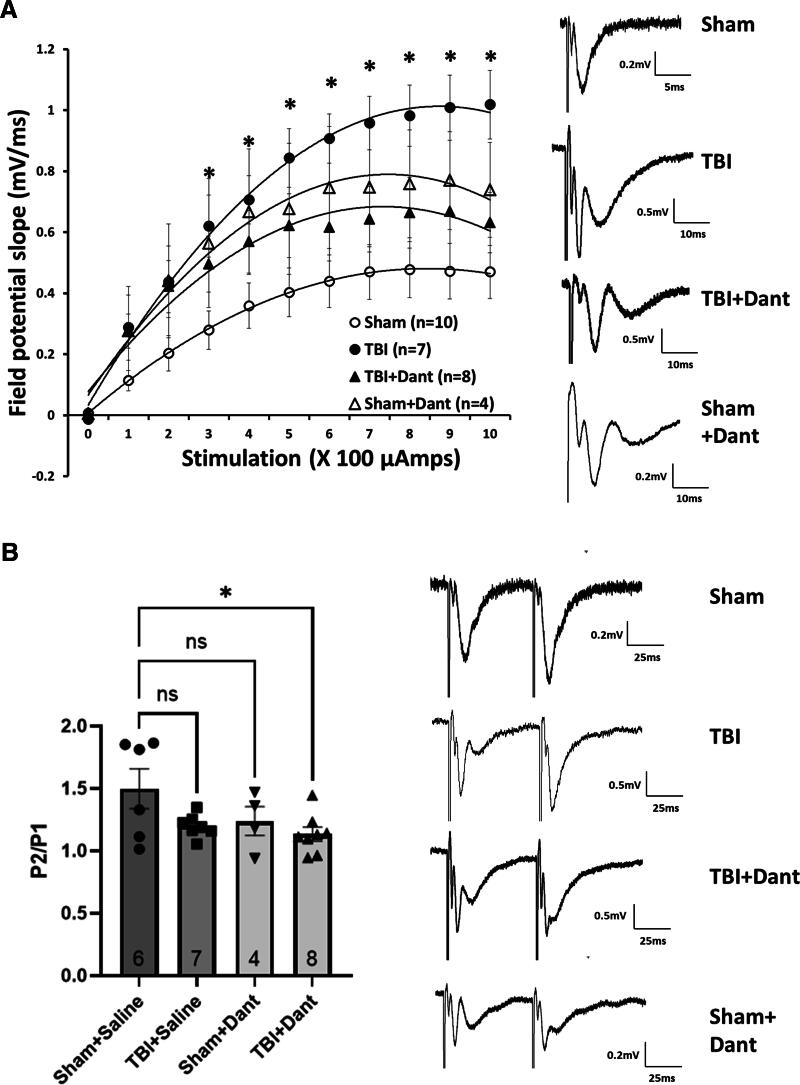
Ryanodex attenuates persistent single mild TBI mediated increases in hippocampal synaptic strength. **(A)** Input-output curves for hippocampal CA1 field potential slopes from sham TBI + saline, TBI + saline, Sham TBI + Ryanodex (Dant) and TBI + Ryanodex (Dant) treatment groups. Field potentials were obtained using CA3-CA1 Schaffer collateral stimulation levels ranging from 0–1000 µA. Hippocampal field potentials obtained from the TBI + saline group had a significantly higher slope at stimulation levels from 300 µA through 1000 µA, an effect that was attenuated in the TBI + Ryanodex (Dant) group. Right, representative hippocampal field potential traces for all treatment groups. **(B)** TBI did not significantly affect the paired-pulse ratio of hippocampal field potentials, a measure of presynaptic release probability. Right, representative paired-pulse field potential traces for all treatment groups. (*p < 0.01. ns, not significant). TBI, traumatic brain injury.

Electrophysiological recordings in hippocampal brain slices were used to monitor changes in synaptic strength due to a single TBI, with and without Ryanodex treatment. Rats were divided into Sham + Saline, TBI + Saline, Sham + Ryanodex and TBI + Ryanodex groups. fEPSPs resulting from stimulation of CA3-CA1 Schaffer Collaterals were measured using the initial slope function of the fEPSP as a measure of synaptic output. Examination of the input-output function of fEPSPs revealed that TBI resulted in significant potentiation at hippocampal synapses throughout a range of stimulation intensities compared to the sham group, an effect that was attenuated by Ryanodex treatment. A two-way repeated-measures ANOVA was significant for effects of TBI treatment group (F (3, 21) = 3.01, *p* = 0.05), and was also significant for stimulation intensity (F (2.20, 46.24) = 69.40, *p* < 0.0001) and treatment group versus stimulation-intensity (F (6.60, 46.24) = 2.48, *p* = 0.03). Post hoc analysis revealed a significant difference between the Sham + Saline and Sham + TBI groups, but not between the Sham + Saline and TBI + Ryanodex or between Sham + Saline and Sham + Ryanodex groups. Ryanodex effects on TBI were significant at 300 μA (*p* = 0.046), 400 μA (*p* = 0.025), 500 μA (*p* = 0.02), 600 μA (*p* = 0.016), 700 μA (*p* = 0.016), 800 μA (*p* = 0.028), 900 μA (*p* = 0.020) and 1,000 μA (*p* = 0.016), indicating that Ryanodex treatment attenuated TBI effects at these stimulation intensities (Fig. 3A). To determine whether the increased synaptic responses in the TBI group reflected presynaptic changes, we used the ratio of paired-pluses, obtained using a 50 ms interstimulus interval, measured as the slope of the second fEPSP divided by the slope of the first fEPSP, as a relative measure of neurotransmitter release probability. A one-way ANOVA was not significant for treatment group (F (3, 25) = 1.57, *p* = 0.22), with *post hoc* analysis showing no significant of TBI on the paired-pulse ratio (*p* = 0.36), when compared to sham (Fig. 3B), suggesting that the synaptic potentiation observed after TBI did not have a presynaptic component, a finding which is in agreement with a previous, similar study.^14^

### Ryanodex prevents mTBI-mediated increases in CA1 pyramidal cell excitability

**FIG. 4.**
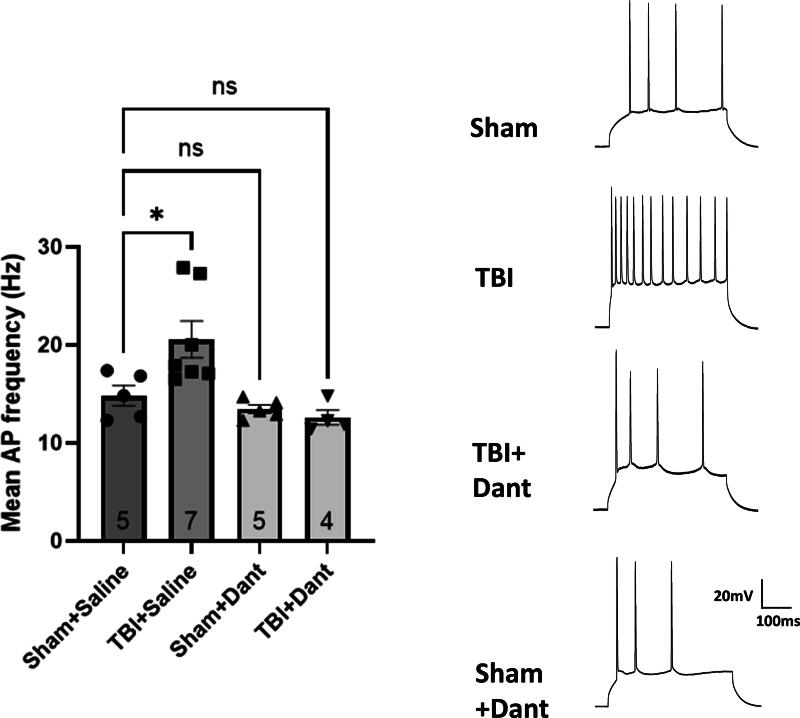
Ryanodex attenuates persistent single mild TBI mediated increases in hippocampal pyramidal cell excitability. (Left) Mean action potential frequency in hippocampal CA1 pyramidal neurons was significantly increased in the TBI + saline group, but not TBI + Ryanodex (Dant), when compared to the sham TBI + saline group. (Right) Trains of action potentials elicited in CA1 pyramidal neurons in response to current injection and depolarization, using whole-cell patch recording, from all treatment groups (*p < 0.05. ns, not significant). TBI, traumatic brain injury.

Hippocampal CA1 pyramidal cell excitability was measured in whole-cell patch recording mode, using positive current injection to depolarize the cell membrane, resulting in a train of action potentials. Excitability was measured as the mean action potential frequency. One-way ANOVA was significant for treatment groups (*F* (3,17) = 7.34, *p* = 0.002), and *post hoc* analysis revealed that the mean action potential frequency was significantly higher in the TBI group compared to the sham group (*p* = 0.02), but that this effect was attenuated when TBI was followed by Ryanodex (*p* = 0.62) (Fig. 4), suggesting that Ryanodex treatment following TBI, mitigates TBI effects on cellular excitability.

### Ryanodex prevents mTBI-mediated increases in CA1 pyramidal cell input resistance

**FIG. 5.**
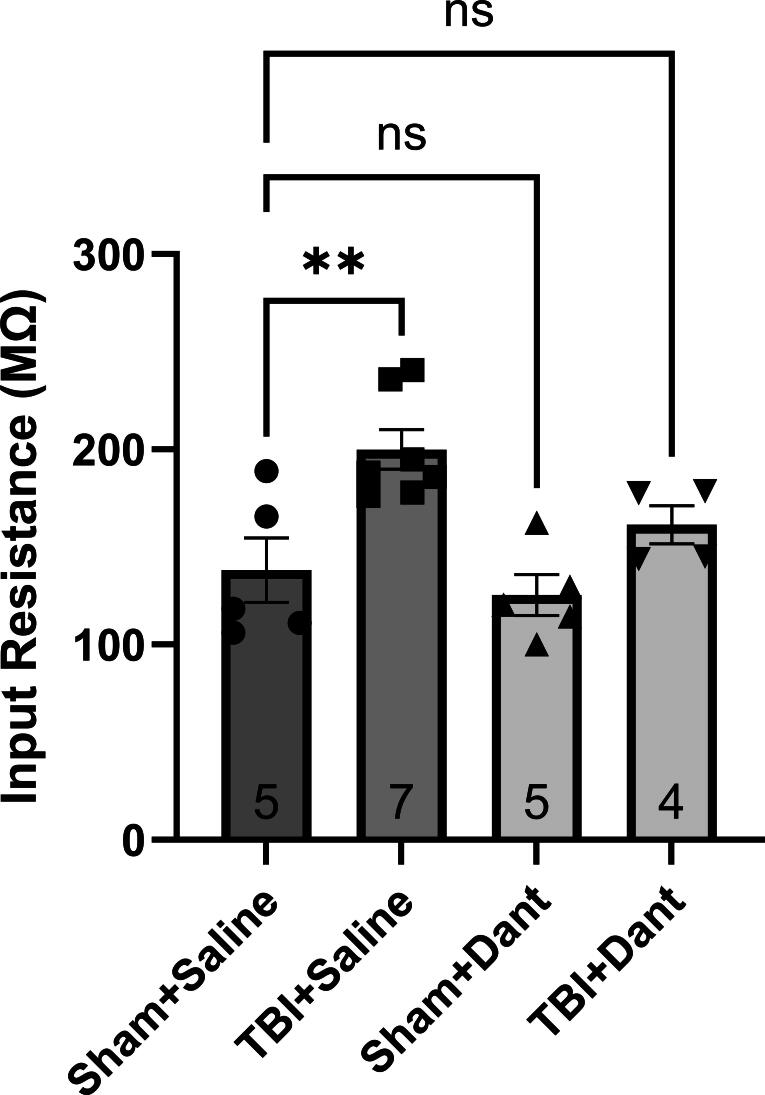
Ryanodex attenuates persistent single mild TBI mediated increases in hippocampal pyramidal cell input resistance. The calculated input resistance in hippocampal CA1 pyramidal neurons was significantly increased in the TBI + saline, but not TBI + Ryanodex (Dant), when compared to the sham TBI + saline group. (**p < 0.05). TBI, traumatic brain injury.

For effects of TBI on input resistance in hippocampal CA1 pyramidal neurons, a possible underlying mechanism for changes in intrinsic excitability resulting from TBI (see above), one-way ANOVA was significant for treatment groups (F (3, 17) = 8.46, *p* = 0.001), and *post hoc* analysis revealed that input resistance was significantly higher in the TBI group compared to sham (*p* = 0.004), and that this effect was attenuated when TBI was followed by Ryanodex (*p* = 0.47) (Fig. 5), suggesting that Ryanodex treatment following TBI, mitigates TBI effects on input resistance. Input resistance of the cell is an intrinsic property that is strongly linked to cell membrane excitability, with increases in input resistance correlating with increases in action potential firing. Given the correlation between effects of TBI, observed here, on cell input resistance on intrinsic cell excitability, as well as their attenuation by Ryanodex, stabilization of RyR function may be beneficial in preventing TBI effects on cell input resistance and excitability.

## Discussion

TBI is a significant public health concern with lasting neurological consequences of even a single impact. This study provides compelling evidence that Ryanodex, an allosteric RyR modulator, mitigates persistent hippocampal hyperexcitability following a single mild TBI. These findings highlight its potential as an acute neuroprotective intervention, addressing an urgent and unmet need in TBI treatment.

A key discovery in this study is the ability of Ryanodex to effectively reverse the pathophysiological synaptic and cellular changes induced by TBI, possibly due to an ability to stabilize intracellular Ca^2+^ dynamics. Previous research has identified RyR-related Ca^2+^ dysregulation as a possible driving factor in neurodegenerative diseases such as AD and CTE.^1,24,25^ Our findings demonstrate that acute intervention targeting RyRs can mitigate TBI-induced hippocampal dysfunction, providing a novel therapeutic avenue for early treatment. One of the interesting findings of this study, was the ability of acute and sub-acute Ryanodex treatment to attenuate hippocampal synaptic potentiation mediated by TBI. While the underlying mechanism for the effects of TBI is still unclear, as Ryanodex was administered as a single dose shortly after impact, and for another two days thereafter, its persistent effects may have been due to stabilization of RyR function, during the acute and subacute phases of TBI. Ryanodex, when given as a subcutaneous injection in mice, results in detectable brain levels of dantrolene after 20 min,^26^ with detectable brain levels persisting for up to 180 min after intranasal administration^27^ and although we did not measure plasma and brain dantrolene, we assume similar pharmacokinetics after intravenous injection. Ryanodex, when given to sham operated rats, did not have the effect of reducing synaptic transmission, indicating that it’s effects overall may be that of a synaptic stabilizer, not inhibitor of RyR function,^28^ this ability to ‘normalize’ synaptic transmission may open up its possibilities as a treatment for diseases of hippocampal synaptic depression, as well as potentiation, for example in animal models of AD, where hippocampal synaptic depression is observed.^29^

**FIG. 6.**
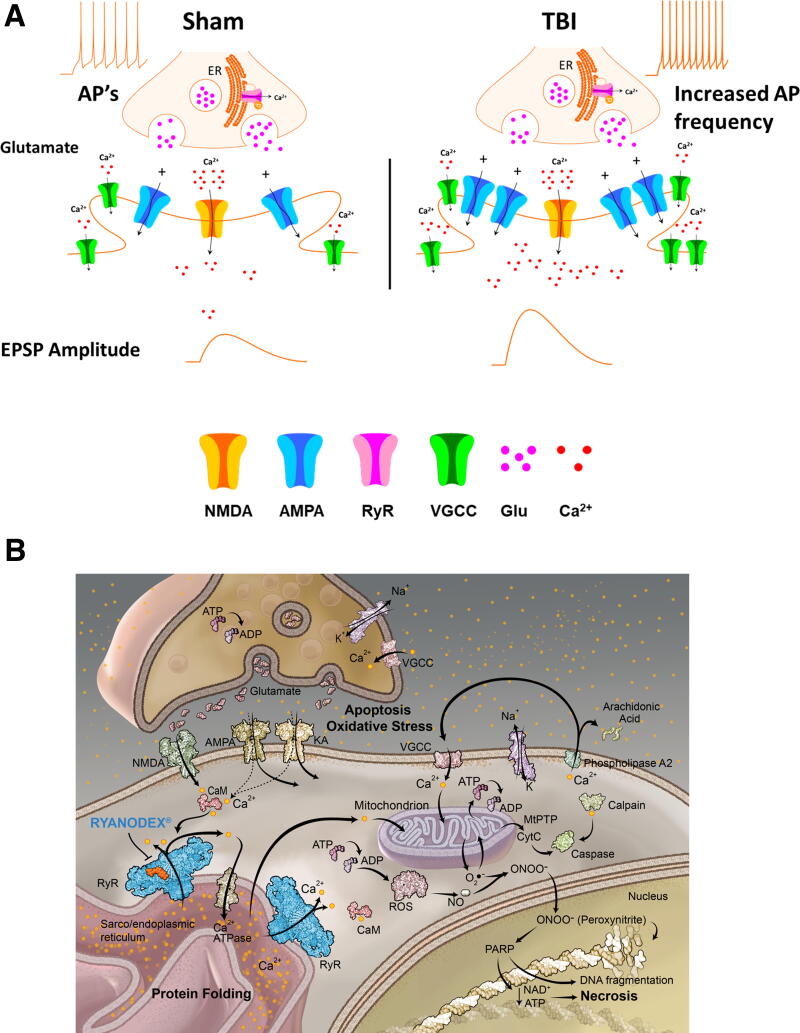
A single mild TBI results in increased hippocampal excitability, both at the synaptic and cellular level. **(A)** Schematic of effects of TBI on synaptic and cellular excitability. A single TBI resulted in a persistently increased hippocampal field potential (EPSP), and increased intrinsic excitability of CA1 pyramidal neurons, with both effects mitigated by Ryanodex, when given acutely and in the days following the impact. The synaptic changes observed may constitute a form of aberrant synaptic plasticity triggered by Ca^2+^ dysregulation arising acutely after impact, including increased postsynaptic AMPA receptor expression. **(B)** Schematic of cellular effects of Ryanodex, involving inhibition/stabilization of RyRs. Stabilization of RyR function by Ryanodex may ameliorate ER stress and Ca^2+^ dysregulation, resulting from acute cellular effects of TBI. EPSP, excitatory postsynaptic potential; ER, endoplasmic reticulum; TBI, traumatic brain injury; RyR, ryanodine receptor; AMPA, Alpha-amino-3-hydroxy-5-methyl-4-isoxazolepropionic acid.

The mechanisms underlying post-concussive cellular events are complex, but they likely involve well-established processes linked to Ca^2+^ dysregulation. Previous studies have reported that Ca^2+^ dyshomeostasis following TBI contributes to synaptic hyperexcitability and neuronal dysfunction.^14,16^ Elevated cytosolic Ca^2+^, increased VGCC activity, and impaired Ca^2+^ buffering mechanisms are key features observed in hippocampal neurons after TBI.^14,15^ After an impact, extracellular glutamate surges activate NMDA receptors, further exacerbating Ca^2+^-mediated synaptic alterations.^30^ These disruptions may result in aberrant synaptic plasticity (see Fig. 6A), where concurrent pre- and postsynaptic activation results in pathological synaptic potentiation. As RyRs are located both pre- and postsynaptically (Fig. 6A and B),^7,31^ their activation may contribute to this dysregulated plasticity, and while we did not see any persistent effects of TBI on the paired-pulse ratio, this does not rule out a role for presynaptic RyRs in the acute and subacute effects of TBI. Postsynaptically, Ryanodex effectively reduces glutamate excitotoxicity *in vitro*,^25^ possibly due to decreased Ca^2+^ effects of NMDA receptor activation,^32^ resulting in decreased RyR activation and overall Ca^2+^ stabilization, a mechanism which may also play a role in the attenuation of the synaptic effects of TBI seen here.

In addition to its effects on hippocampal synaptic function, Ryanodex also prevented TBI-induced increases in CA1 pyramidal cell excitability and input resistance. These findings align with previous observations in rodent models of AD and other neurodegenerative diseases.^33,34^ Input resistance of the cell is an intrinsic property that is strongly linked to cell membrane excitability, with increases in input resistance correlating with increases in action potential firing. Given the correlation between effects of TBI, observed here, on cell input resistance on intrinsic cell excitability, as well as their attenuation by Ryanodex, stabilization of RyR function may be beneficial in preventing TBI effects on cell input resistance and excitability. Although there is no direct link for RyR function to cell excitability, one underlying mechanism may involve RyR interaction with key ion channels such as the Kv2.1 delayed rectifier potassium channel, which plays a crucial role in neuronal excitability, and are susceptible to oxidative modifications following TBI.^35^ Oxidation-induced inhibition of Kv2.1 has been linked to neurotoxicity,^36,37^ and its stabilization may be a secondary mechanism through which Ryanodex exerts its neuroprotective effects. As Kv2.1 channels form functional clusters with RyRs,^38^ Ryanodex stabilization of RyRs may inhibit mTBI-related effects on cell excitability through this interaction. In addition, stabilization of RyR function using dantrolene sodium results in excitation of hippocampal pyramidal neurons, due to a reduction in action potential afterhyperpolarization,^39^ an effect that was accompanied by an increase in the input resistance. Whether these effects were due to RyR stabilization or inhibition by Ryanodex is still debatable, but similar effects were not observed upon inhibition of RyRs using ryanodine,^40^ indicating a possible different mode of action for Ryanodex.

While our findings are that single or TBI results in hippocampal synaptic potentiation, multiple studies have shown synaptic depression resulting from TBI,^41–43^ or at least impairment of acute plasticity.^42,44,45^ While inconsistency of experimental results is not new, reasons for the different findings seen here and elsewhere may be due to differences in the TBI model used, due either to species differences (rat vs mouse), mode of injury (latera fluid percussion in open head, blast injury, closed-head CCI),^14,41,42,44^ time of assessment (days to weeks after injury), and number of injuries (single vs. repeated).^14,20,46^ Because of these different protocols, different outcomes are to be expected; however, the findings of this study are in agreement with a previous study, carried out using the same protocol,^14^ thus illustrating the importance of the protocol as a factor in the experimental outcome. Although our findings are not in total agreement with other TBI studies, our findings are consistent with the neuroprotective effects of dantrolene sodium observed in another closed-head rat model of TBI,^47^ and this is encouraging for the future use of dantrolene as a treatment for TBI.

Pathogenic hyperphosphorylated tau is a hallmark of various neurodegenerative disorders, including TBI, CTE, and AD.^1^ Although hyperphosphorylated tau has been observed in single and repeated TBI cases,^14,48^ we did not detect an increase in our study. This may be due to the transient nature of tau phosphorylation, as its half-life is approximately 10 days,^49^ and our tissue harvest occurred at day 30. Nonetheless, the potential link between hyperexcitability and tau pathology remains an area of interest, as previous research suggests that neuronal hyperexcitability precedes tau deposition in AD models^50^ and clinically normal older adults.^51^ Future long-term studies will be essential to establish this relationship in TBI.

Beyond its immediate effects on hippocampal function, our findings have broader implications for long-term neurological health. TBI is increasingly recognized as a precursor to neurodegenerative conditions, where aberrant Ca^2+^ signaling plays a central role in disease progression.^1,9^ If Ryanodex can prevent or attenuate these pathological cascades, it could transform the treatment paradigm for the patients with TBI, particularly those at high risk for neurodegeneration. Given that Ryanodex is already FDA-approved for malignant hyperthermia, and other conditions, with minimal side-effects, its established safety profile may facilitate translation into clinical trials for TBI, expediting the development of the first targeted pharmacological treatment for acute brain injury.

## Conclusions

Overall, our findings indicate that RyR channels may be an important therapeutic target for mitigating the neurological effects of TBI. The ability of Ryanodex to modulate RyR activity may offer a promising approach for both acute and long-term intervention following brain injury. Additionally, this study highlights the broader applicability of RyR-targeted therapies in conditions such as AD and CTE. Future research should focus on optimizing treatment protocols and further exploring the molecular mechanisms underlying the protective effects of Ryanodex. With continued investigation, this approach could lead to groundbreaking advancements in the treatment of TBI, offering hope to millions affected by brain injury each year.

## Transparency, Rigor, and Reproducibility Statement

No study and analysis plan were registered prior to beginning data collection. A sample size of 6 per treatment group was planned for electrophysiology based on an assumption of a minimum of 1 field recording or 1 whole-cell patch recording per rat from a total of 12 rats per group, for 3 groups. While a sample size of 6 per group was also planned for immunoblotting, we only obtained 3 samples each from the 2 non-drug groups. For the experiments, 44 rats were purchased, 42 survived the experimental manipulations, and complete data were obtained on all 42. Rats were randomly assigned to groups, based on a random number generator. Electrophysiology and immunoblotting were performed by investigators blinded to treatment group. All closed-head cortical impacts were performed between 10am-noon, using a Leica Impact one, which was attached to a stereotaxic frame, to ensure accuracy and reproducibility of impacts. Antibodies used were recombinant rabbit monoclonal antibodies from Abcam, to ensure consistency and reproducibility of antibody specificity from lot to lot. All Ryanodex doses used were from the same lot number, supplied as the clinical formulation, from Eagle Pharmaceuticals. No replication or external validation studies have been performed or are planned/ongoing at this time to our knowledge. Data from this study will be made available upon request. No specific analytical code was used for image quantitation. Samples from each of the experimental groups used for immunoblotting are available for future analyses on request. This paper will be published under a Creative Commons Open Access license *(CC-BY 4.0)*, and upon publication will be freely available at https://home.liebertpub.com/publications/neurotrauma-reports/657.

## Data Availability

Data will be made available upon reasonable request.

## Abbreviations Used

AD: Alzheimer’s disease
aCSF: Artificial cerebrospinal fluid
AMPA: Alpha-amino-3-hydroxy-5-methyl-4-isoxazolepropionic acid
ANOVA: Analysis of variance
Ca2^+^: Calcium
CCI: Controlled cortical impact
CTE: Chronic traumatic encephalopathy
EPhys: Electrophysiology
EPSP: Excitatory postsynaptic potential
ER: Endoplasmic reticulum
FDA: United States Food and Drug Administration
fEPSP: Field excitatory postsynaptic potential
GFAP: Glial fibrillary acidic protein
HEPES: 4-(2-hydroxyethyl)-1-piperazineethanesulfonic acid
IgG: Immunoglobulin G
i.v.: Intravenous
LDS: Lithium dodecyl sulfate
mTBI: Mild traumatic brain injury
NMDG: N-methyl-d-glucamine
PVDF: Polyvinylidene difluorid
RyR: Ryanodine receptor
SDS-PAGE: Sodium Dodecyl Sulfate Polyacrylamide Gel Electrophoresis
TBI: Traumatic brain injury
TBST: Tris-Buffered Saline with Tween 20
VGCC: Voltage gated calcium
